# Integrative meta-omics in Galaxy and beyond

**DOI:** 10.1186/s40793-023-00514-9

**Published:** 2023-07-07

**Authors:** Valerie C. Schiml, Francesco Delogu, Praveen Kumar, Benoit Kunath, Bérénice Batut, Subina Mehta, James E. Johnson, Björn Grüning, Phillip B. Pope, Pratik D. Jagtap, Timothy J. Griffin, Magnus Ø. Arntzen

**Affiliations:** 1 Faculty of Chemistry, Biotechnology and Food Science, Norwegian University of Life Sciences (NMBU), P.O. Box 5003, 1432 Ås, Norway; 2 Department of Biochemistry, Biophysics and Molecular Biology, University of Minnesota, Minneapolis, MN 55455 USA; 3 Bioinformatics Group, Department of Computer Science, University of Freiburg, Freiburg, Germany; 4 Minnesota Supercomputing Institute, University of Minnesota, Minneapolis, MN 55455 USA; 5 Faculty of Biosciences, Norwegian University of Life Sciences (NMBU), P.O. Box 5003, 1432 Ås, Norway

**Keywords:** Integrated meta-omics, Metagenomics, Metatrascriptomics, Metaproteomics, Galaxy, Bioinformatics

## Abstract

**Background:**

‘Omics methods have empowered scientists to tackle the complexity of microbial communities on a scale not attainable before. Individually, omics analyses can provide great insight; while combined as “meta-omics”, they enhance the understanding of which organisms occupy specific metabolic niches, how they interact, and how they utilize environmental nutrients. Here we present three integrative meta-omics workflows, developed in Galaxy, for enhanced analysis and integration of metagenomics, metatranscriptomics, and metaproteomics, combined with our newly developed web-application, ViMO (Visualizer for Meta-Omics) to analyse metabolisms in complex microbial communities.

**Results:**

In this study, we applied the workflows on a highly efficient cellulose-degrading minimal consortium enriched from a biogas reactor to analyse the key roles of uncultured microorganisms in complex biomass degradation processes. Metagenomic analysis recovered metagenome-assembled genomes (MAGs) for several constituent populations including *Hungateiclostridium thermocellum, Thermoclostridium stercorarium* and multiple heterogenic strains affiliated to *Coprothermobacter proteolyticus*. The metagenomics workflow was developed as two modules, one standard, and one optimized for improving the MAG quality in complex samples by implementing a combination of single- and co-assembly, and dereplication after binning. The exploration of the active pathways within the recovered MAGs can be visualized in ViMO, which also provides an overview of the MAG taxonomy and quality (contamination and completeness), and information about carbohydrate-active enzymes (CAZymes), as well as KEGG annotations and pathways, with counts and abundances at both mRNA and protein level. To achieve this, the metatranscriptomic reads and metaproteomic mass-spectrometry spectra are mapped onto predicted genes from the metagenome to analyse the functional potential of MAGs, as well as the actual expressed proteins and functions of the microbiome, all visualized in ViMO.

**Conclusion:**

Our three workflows for integrative meta-omics in combination with ViMO presents a progression in the analysis of ‘omics data, particularly within Galaxy, but also beyond. The optimized metagenomics workflow allows for detailed reconstruction of microbial community consisting of MAGs with high quality, and thus improves analyses of the metabolism of the microbiome, using the metatranscriptomics and metaproteomics workflows.

## Introduction

Microbial communities have a tremendous impact on Earth’s ecosystems. An example is the marine microbiome, which is responsible for > 50% of the produced oxygen on the planet [1]. The microorganisms historically promoted the adjustment from freshwater to the terrestrial environment for plants [2] and bacteria still today regulate the growth and development of the terrestrial flora by symbiosis, for example promoting growth by nitrogen fixation or plant hormone production [3]—dynamic and highly adaptable processes that are influential to microbial communities and their hosts alike [4, 5]. Similarly, in humans, microbial communities may affect the toxicity of drugs, modulate disease progression, and promote health. It is of great importance to increase our understanding of such microbiomes, their composition and interplay, as well as factors for perturbation, stability, and development [6, 7]. Ideally, such understanding may spark the development of new personalized medical treatments for improving life quality and addressing the climate crisis, specifically by curbing the emissions of methane from wetlands or ruminating animals [8, 9] and nitrous oxide from agriculture [9, 10].

Meta-omics technologies, alongside environmental measurements, allow researchers to infer the complex network of a microbiome and its relations with the environment and host, offering a putative picture of their metabolism in their natural habitat [11, 12]. With metagenomics, we analyse the total DNA of the microbial community using shotgun sequencing [11, 13, 14], and this technology provides information about the potential physiological function and regulation of the genes in microbial communities [11, 15, 16]. Modern tools for read assembly allow for the retrieval of both known and novel organisms by overcoming challenges such as size and complexity of metagenomic data, as well as difficulties in accuracy and contiguity of metagenome assemblies [17]. This have resulted in larger and less fragmented assemblies and hence better quality of metagenome-assembled genomes (MAGs) [18]. Remarkably, in some samples, species-resolution can be achieved during the binning process, allowing for reconstruction of metabolic pathways for individual MAGs [19]. Further, metatranscriptomics aims to analyse the entire set of active gene transcripts in the microbial community as well as calculate their (relative) abundances and thus capture perturbation, environmental changes, and dynamics [14, 16]. Using high-throughput sequencing, transcripts of microorganisms are detected, and either analysed on their own, or preferably, mapped to the metagenomics data, including MAGs, which enables the identification and quantification of active metabolic pathways [14]. Further evidence is provided by metaproteomics, which identifies and quantifies of the entire set of proteins in the microbial community, both intra- and extra-cellular [11, 16]. Metaproteomics in combination with metagenomics allows both for targeted identification of sample-specific microorganisms, and also for the identification of proteins not present in publicly available sequence repositories such as UniProt or RefSeq. This in turn might enhance our understanding of known signalling pathways or possibly act in the discovery of new metabolic pathways [20], as well as detect the presence of active novel microbial members within the community.

Due to a rapid improvement of algorithms within the meta-omics field, analysing meta-omics data requires a constant update and evaluation of computational tools. Currently, hundreds of tools are available for the analysis of meta-omics data, and it can be challenging to select the right tool and parameters for a given dataset. Meanwhile, the popularity of user-friendly interfaces attached to compute resources with pre-installed software packages, like Anvi’o [21] for metagenomics and metatranscriptomics, iMetalab [22] for metaproteomics, and Galaxy for multi-omics [23, 24], are on the rise, particularly because they enable advanced bioinformatic analysis without the need for programming/scripting. In the Galaxy platform, various tools can be chained together in a sequential manner into a workflow and shared between developers and users for further data-based optimization and reproducibility [25]. A common workflow for metagenomics within Galaxy is ASaiM [26] with taxonomic and functional analysis of metagenomics shotgun data, which was further extended to include metatranscriptomics analysis in the ASaiM-MT workflow [27]. However, while ASaiM and ASaiM-MT offer in-depth microbial analysis, it currently does not support the analysis of MAGs or the full integration between the different omics disciplines.

In this study, we applied commonly used omics tools within the Galaxy framework to generate workflows for metagenomics (MetaG), metatranscriptomics (MetaT), and metaproteomics (MetaP). We made the workflows integrative, so that MAGs recovered in the MetaG workflow makes the reference for mapping both transcriptomic reads and proteomic mass spectra. The workflows were applied on a highly efficient cellulose-degrading minimal consortium enriched from an industrial biogas reactor in Fredrikstad, Norway to analyse the key roles of uncultured microorganisms in complex biomass degradation processes [28]. To enhance the multi-levelled data interpretation and exploration, we developed an interactive R-Shiny-based web-application, ViMO (Visualizer for Meta-Omics), where the data can be explored in more detail.

## Methods

### Samples

The microbial community called SEM1b studied/utilized in this work was enriched from a thermophilic biogas reactor operated on municipal food waste (Frevar) and manure in Fredrikstad, Norway, and has previously been described in detail, including metagenomics, metatrascriptomics and metaproteomics analysis across nine time points spanning over 43 h post inoculation [28, 29]. In brief, using an inoculate from a lab-scale reactor, we performed a serial dilution to extinction experiment to simplify and enrich the community for growth on Norwegian Spruce as carbon source at 65 °C. DNA was collected by Phenol–Chloroform extraction of 6 mL sample and a library was prepared with the TrueSeq DNA PCRfree-protocol prior to sequencing on an Illumina HiSeq3000 platform (Illumina Inc) with paired-ends (2 × 125 bp) [28, 29]. For metatranscriptomics analysis, mRNA was extracted in triplicates (A, B, and C) with the RNeasy mini kit (Protocol2, Qiagen, USA) followed by DNA and small RNAs removal (such as tRNA) with lithium chloride precipitation solution (ThermoFisher Scientific) according to manufacturer’s recommendation. The enriched mRNA was amplified with the MessageAMP II-Bacteria Kit (Applied Biosystems, USA) and sequenced on an Illumina HiSeq3000 platform with paired-ends (2 × 125 bp). Proteins were extracted chemically and mechanically using FastPrep24 in triplicates and subsequently reduced, alkylated and in-gel digested with trypsin. The mass spectrometry analysis of the peptides was performed using nanoLC-MS/MS system consisting of a Dionex Ultimate 3000 UHPLC (ThermoScientific, Germany) connected to a Q-Exactive hybrid quadrupole-orbitrap mass spectrometer (ThermoScientific, Germany). For this study, we used the metagenomics data from the abovementioned SEM1b community, as well as a subset of the metatranscriptomics and metaproteomics data, including triplicates from three time points (13, 23, 38 h) after inoculation [28, 29].

## Implementation, results and discussion

In this study we used common tools already present within the Galaxy ToolShed (https://toolshed.g2.bx.psu.edu/), as well as incorporated additional tools (dRep, CheckM, CoverM, BAT/CAT) to facilitate multi-omics analysis of microbiomes at a level not possible in Galaxy previously. The newly implemented dRep selects MAGs with the best quality in the genome set improving the pathway analysis of each MAG with functional annotation tools and the recently added KOFamScan annotations. The quality for these MAGs in the workflow can be assessed with CheckM and their genome mapped back to the metagenome raw files using CoverM. Tools for meta-omics were then chained to generate three separate workflows for (1) metagenomic assembly, binning, and functional annotation (MetaG), (2) metatranscriptomics (MetaT), and (3) metaproteomics (MetaP). Although separate, the workflows are designed to be integrative so that the MAGs recovered from MetaG make the foundation for mapping both the transcriptomic reads and the proteomic spectra onto their predicted genes. The tools included in the three pipelines are listed in Table 1.

**Table 1:** List of software in the MetaG, MetaT, MetaP workflows

Workflow	Software version	References/webpage
*MetaG*		
Trimming	Trim Galore! (Galaxy version 0.6.7 + galaxy0)	(https://www.bioinformatics.babraham.ac.uk/projects/trim_galore/)
Quality control	FastQC (Galaxy version 0.73 + galaxy0)	(https://www.bioinformatics.babraham.ac.uk/projects/fastqc/)
Assembly	MEGAHIT (Galaxy version 1.1.3.5)	[30]
Assembly quality	metaQUAST (Galaxy version 5.2.0 + galaxy0)	[31]
Binning	MaxBin2 (Galaxy version 2.2.7 + galaxy3)	[19]
Dereplication*	dRep (Galaxy version 3.2.2 + galaxy0)	[32]
Genome quality assessment	CheckM lineage_wf (Galaxy Version 1.2.0 + galaxy0)	[33]
Read coverage	CoverM-GENOME (Galaxy Version 0.2.1 + galaxy0)	(https://github.com/wwood/CoverM)
Read coverage	CoverM-CONTIG (Galaxy Version 0.2.1 + galaxy0)	(https://github.com/wwood/CoverM)
Bin annotation	CAT bins (Galaxy version 5.0.3.0)	[34]
Gene prediction	FragGeneScan (Galaxy version 1.30.0)	[35]
CAZyme annotation	Hmmscan (Galaxy version 0.1.0) with dbCAN-HMMdb-V10	[36–38]
KOfam annotation	KofamScan (Galaxy version 1.3.0 + galaxy1)	
Functional annotation	Interproscan (Galaxy version 5.0.0)	[39]
*MetaT*		
Trimming	Trimmomatic (Galaxy version 0.38.1)	[40]
Quality control	FastQC (Galaxy version 0.73 + galaxy0	(https://www.bioinformatics.babraham.ac.uk/projects/fastqc/)
rRNA removal	SortMeRNA (Galaxy version 2.1b.6)	[41]
mRNA quantification and mapping	Kallisto quant (Galaxy version 0.46.0.4)	[42]
*MetaP*		
Protein quantification	MaxQuant (Galaxy version 1.6.3.4)	[43, 44]

*Tools unique for the optimized MetaG workflow

### Workflow for metagenomics and functional annotation (MetaG)

The MetaG workflow provides all the processing steps and parameters to analyze FASTQ files containing the shotgun metagenomics raw data. This multi-step workflow contains data cleaning/trimming, assembly of reads into contigs, binning of contigs into MAGs, as well as taxonomic analysis of the MAGs and functional annotation of all gene products encoded in the MAGs (Table 1).

The MetaG workflow accepts Illumina paired-end FASTQ sequence files (forward and reverse reads) as *input files* (Fig. 1.1). The FASTQ-files can be uploaded to Galaxy via the web interface or using FTP and should be organized as a collection of paired datasets. As *quality control* (Fig. 1.2), we use FastQC (https://www.bioinformatics.babraham.ac.uk/projects/fastqc/) with a Phred threshold of 20 to be aware of occasional nucleotide reading errors or overrepresentation of features, like primers or sequencing adapters. The quality control is followed by a data preprocessing steps, including automatic detection and *trimming* (Fig. 1.3) of adapter sequences by Trim Galore! (https://www.bioinformatics.babraham.ac.uk/projects/trim_galore/). The collection of trimmed paired reads is then split into a list of forward and reversed reads for *co-assembly*. The metagenomic reads are further assembled (Fig. 1.4) into contigs with k-mer sizes of 21, 29, 39, 59, 79, 99, 119, and 141 using MEGAHIT [30]. The quality for assemblies is assessed using metaQUAST [31] (Fig. 1.5) in meta-mode. The contigs are *binned* into MAGs (Fig. 1.6) by MaxBin2 [19] based on an expectation–maximization algorithm with a minimum contig length of 1000. Completeness, contamination, and strain heterogeneity are analyzed using *CheckM* [33] and read coverage using *CoverM* (https://github.com/wwood/CoverM) (Fig. 1.7). Further, *taxonomic annotation* for the MAGs is done with the Bin Annotation Tool [34] (range: 10, fraction: 0.5) (Fig. 1.8). The genomes are individually subjected to *gene prediction* (Fig. 1.9) using the software FragGeneScan [35], which outputs FASTA-files of both nucleotide and protein sequences.

The putative proteins are then *functionally annotated* (Fig. 1.10) using InterProScan [39] with the databases TIGERFAM [45], HAMAP [46], PfamA [47], and Gene Ontology [48], while KoFamScan [49] provides enzyme commission numbers (EC) and annotation from KEGG [50]. For prediction of carbohydrate-active enzymes (CAZymes), the MetaG workflow uses Hidden Markov Models from dbCAN [38], downloaded from https://bcb.unl.edu/dbCAN2/ and used within the software HMMER [51]. To facilitate downstream analyses, we combine all the functional annotations from InterProScan, KoFamScan and dbCAN into one file using a script within the Galaxy implementation of awk to generate a tabular file with one protein per row and the different annotations in individual columns. This file of functional annotation of all gene products in the metagenome, together with the output from taxonomic analysis, is used for more detailed data exploration and interpretation in *ViMO* (Fig. 1.18). Optionally, the putative genes and proteins from FragGeneScan [35] can be manually augmented with strains from public repositories such as NCBI, UniProt or IMG.

**Fig. 1:**
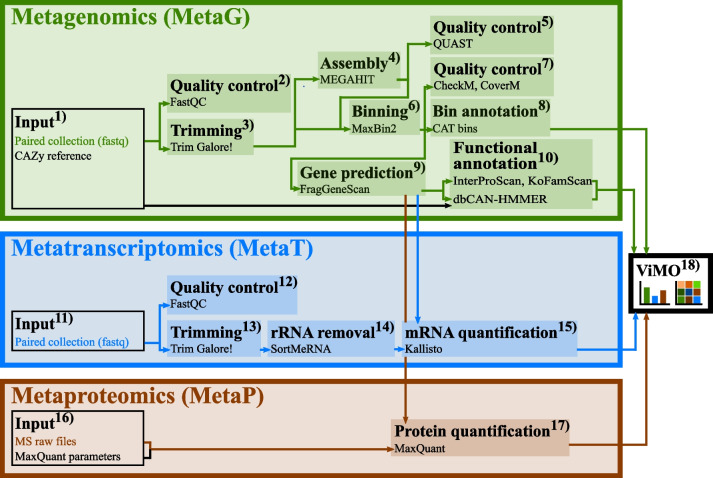
Workflows for meta-omics. The integrated analysis of meta-omics contains a MetaG, MetaT and MetaP workflow. MetaG includes data preprocessing steps with quality control and trimming, followed by assembling, binning and taxonomically annotation of the MAGs. Open reading frames (ORFs) and nucleotide sequences are predicted by FragGeneScan. Functional annotation is performed by InterProScan and dbCAN-HMMER. The predicted ORFs and nucleotide sequences are further used in the MetaP and MetaT workflow; hence, the MetaG serves as the base analysis and the MetaT and MetaP are mapped onto the MetaG. After preprocessing the data and rRNA removal, the predicted nucleotide sequences from the MetaG workflow are used for the mRNA quantification and mapping by Kallisto, as well as for MaxQuant in the MetaP workflow

### Workflow for metatranscriptomics (MetaT)

The MetaT workflow provides all the processing steps and parameters to analyze raw metatranscriptomics paired-end reads. This multi-step workflow contains data cleaning/trimming, RNA filtering, mRNA quantification, and mapping to the predicted genes from the metagenome from the MetaG workflow (Table 1).

As *input files* (Fig. 1.11), the MetaT workflow accepts Illumina FASTQ sequence files (forward and reversed reads), which can be uploaded to Galaxy via web interface and organized as a collection of paired datasets. The workflow includes data preprocessing, where *quality control* (Fig. 1.12) of the sequences is done with FastQC to assess the overrepresentation of features, such as primers or adapters, with a Phred threshold of 20. Adapter sequences are automatically detected and *trimmed* (Fig. 1.13) by Trim Galore!. Sequencing of RNA results in a mixture of coding and non-coding RNA fragments, and the highly abundant ribosomal RNA in the samples are *filtered out* (Fig. 1.14) in order to use only mRNA transcripts for the analysis [52]. Thus, rRNA and tRNA are removed using the software SortMeRNA [41]. This is followed by *mRNA quantification and mapping* (Fig. 1.15). The mRNA quantification is done with the software Kallisto [42], which pseudoaligns mRNA reads onto nucleotide sequences (in this case the predicted genes from FragGeneScan in the MetaG workflow), and is thereby skipping alignment for redundant kmers in the De Bruijn graph from the transcriptome, which saves time while being accurate and sensitive [42]. The outputs from Kallisto, one per sample, are finally joined in order to generate one single file to use in *ViMO* (Fig. 1.18).

### Workflow for metaproteomics (MetaP)

For the MetaP workflow, RAW files from the mass spectrometric analysis are uploaded to Galaxy via the web interface or FTP and organized as a collection list. MaxQuant [43] within Galaxy (version 1.6.17.0) require uploading a file describing the experimental design, i.e., a text-file with a list of all the RAW files and which experiment/biological replicate they belong to (Fig. 1.16). The rest of the parameters can be selected at run-time in Galaxy, including proteolytic cleavage, matching between runs, fixed and variable peptide modifications, and parameters for identification; for this dataset, these are described in Delogu et al. [28]. *MaxQuant* (Fig. 1.17) in Galaxy is then used to identify and quantify proteins by matching MS/MS spectra onto the protein sequences predicted by FragGeneScan [35] in the MetaG workflow (Table 1). The output from MaxQuant (Proteingroups.txt) is used for downstream analysis in *ViMO* (Fig. 1.18). It should be noted that MaxQuant has some limitations with large databases (> 500.000 protein entries), and we are seeking to replace this software with FragPipe in the future versions of this MetaP workflow to scale along the fast growth in metagenomics in recovering hundreds of MAGs from various samples.

## Data integration in ViMO: visualizer for meta-omics

Analyzing and exploring multi-leveled meta-omics data is not a trivial task and requires linking information from metagenomics, such as the presence of specific pathways within selected MAGs, with expression data from transcriptomics and proteomics analysis. This level of data integration is complicated and not practical in spreadsheet applications such as Excel and is thus typically achieved through scripting with Python or R. Preferably, interactive tables and maps would allow data exploration where the user can browse through the catalog of MAGs present in the samples and their metabolisms, while receiving visualizations of expressed genes and functions. This was our motivation for developing ViMO.

ViMO is provided with a script that reads the following output from the MetaGTP workflows and generates a Masterfile and a Contig file for import: (1) All the dereplicated genomes with their contigs, (2) the file containing all putative proteins annotated with functional predictions from InterProScan, dbCAN and KoFamScan, (3) metagenomic coverages of contigs as well as completeness, contamination and strain heterogeneity from CoverM and CheckM, (4) the taxonomic annotations from CAT/BAT, (5) the quantification of mRNA from Kallisto, and (6) the quantification of proteins from MaxQuant. Obviously, ViMO is also functionable with a similar Masterfile generated from a custom workflow, either in Galaxy or elsewhere, e.g., using different software for quantification such as FragPipe [53], as long as the essential columns are present in the final Masterfile; this is described in the help-section of ViMO.

**Fig. 2:**
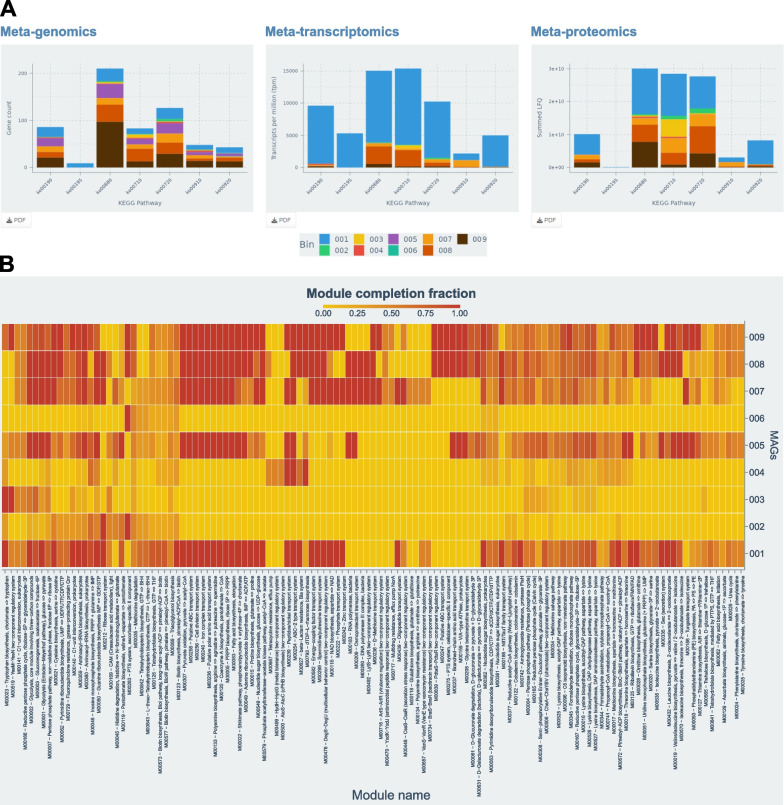
ViMO visualizations. **A **ViMO produces bar plots to visualize the gene counts and abundances of KEGG-pathways in the different bins, here filtered to pathways in energy metabolism. For metagenomics, all timepoints are used, while for metatranscriptomics and metaproteomics, only the first timepoint is shown here and the user can select which sample/timepoint to visualize. In addition, ViMO displays heatmaps with all timepoints within one graph for metatranscriptomics and metaproteomics to visualize temporal changes (data not shown). **B **ViMO calculates the module completion fraction (mcf) for all KEGG modules (x-axis; only a subset displayed here) and MAGs (y-axis) and thus visualize the metabolic potential of each MAG. The set of visible modules can be filtered to selected KEGG pathways for in-depth exploration

**Fig. 3:**
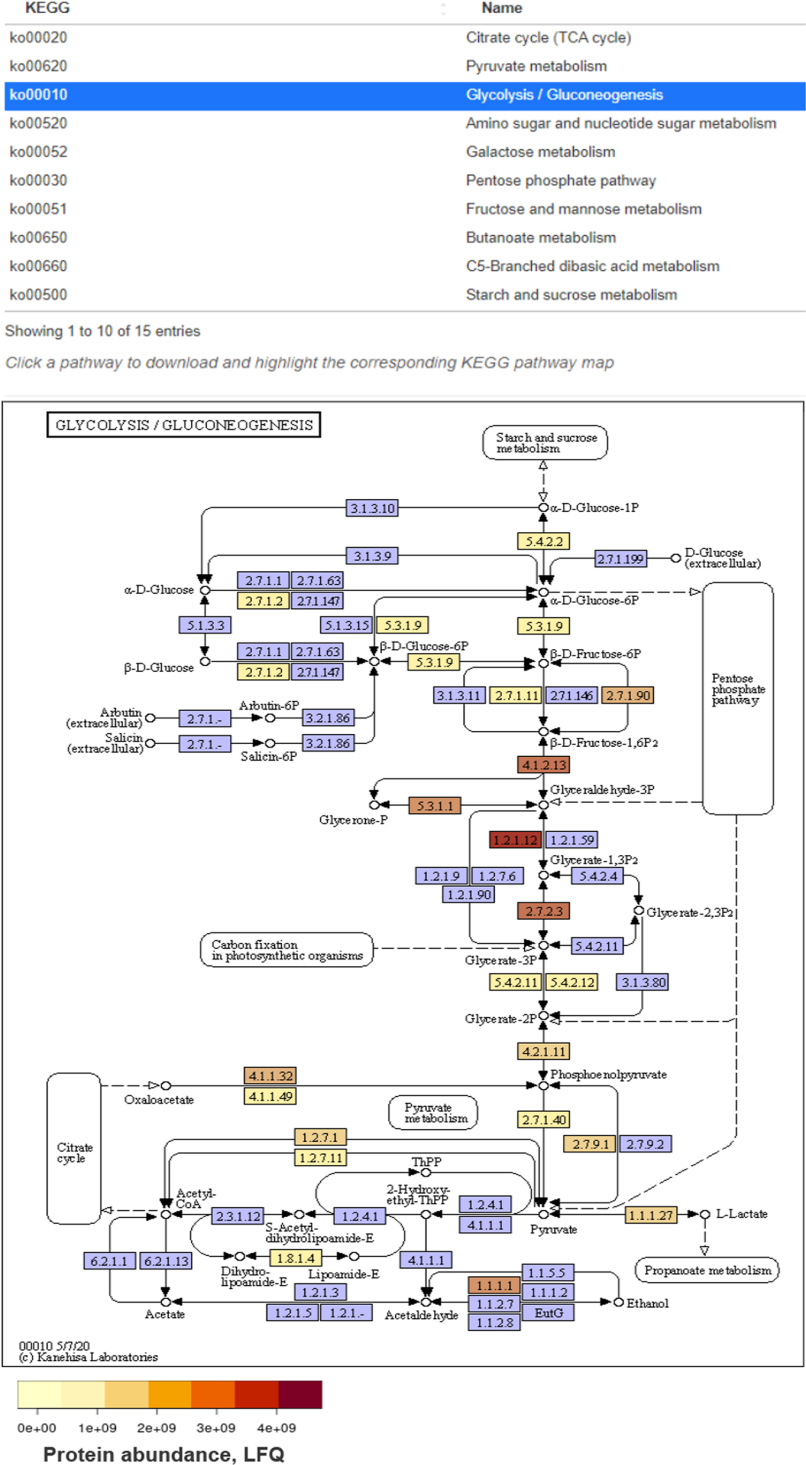
Annotated KEGG-maps. In ViMO, when KEGG-pathways are selected (top, filtered to pathways in carbohydrate metabolism), a KEGG-map is downloaded and annotated with abundances of expressed genes for the selected MAG. Here is shown the Glycolysis/Gluconeogenesis pathway of MAG001, a bacterium from the Tissierellia class in the SEM1b community, annotated with metaproteomic abundances ranging from low-abundant (0 LFQ; light yellow) to high-abundant (4e9 LFQ; dark red); blue enzymes are not detected in the metaproteome for this MAG

Once the files are loaded, ViMO provides four core analyses. (1) MAGs, an overview of all detected MAGs including counts of contigs and genes, contamination, completeness and taxonomy, as well as a figure of %GC versus metagenomic coverage to illustrate the coherence within each MAG. (2) CAZy, an overview of all detected CAZymes including carbohydrate esterases (CEs), glycosyl transferases (GTs), glycoside hydrolases (GHs), polysaccharide lyases (PLs), carbohydrate binding domains (CMBs), auxiliary activities (AAs) and components of cellulosomes, with their counts and abundances at both mRNA and protein level. Heatmaps allow for visualization of temporal changes between samples, if applicable in the experimental design. (3) KEGG, an overview of all genes with a KEGG annotation, sorted and selectable into KEGG pathways, with counts and abundances at both mRNA and protein level (Fig. 2A). ViMO allows filtering down to a specific pathway and downloads KEGG-maps and highlight the detected enzymes within the pathways with colors representing abundance, at both mRNA and protein level (Fig. 3). This allows detection of highly expressed pathways within the microbial community and in which MAGs they are most abundant. While this is possible to retrieve through the standard KEGG web-interface (KEGG Mapper [50]), one would have to copy all the proteins and abundances into the web-interface manually and for one MAG at the time, while ViMO retrieves this information automatically while the user browses through the MAGs. (4) KEGG-Modules, calculate the module completion fraction (mcf) for all KEGG-modules in all MAGs and visualize the metabolic potential of each MAG in a heatmap (Fig. 2B). This can optionally be filtered to lower-level KEGG categories. The powerful KEGG modules network allows for inspecting the completeness, meaning the presence of the complete set of enzymes required for a given metabolic reaction and was implemented in ViMO using the R-package MetQy [54]. Alternatively, similar heatmaps can be generated with the KEGGDecoder software [55]; however, here this is done automatically within ViMO and with interactive filtering options.

In terms of limitations and guidelines for best usage, ViMO works best with meta-omics datasets containing up to ~ 50 MAGs/ ~ 150.000 genes due to the extensive plotting and interactivity. Although we have successfully assessed its functionality with larger datasets of > 250 MAGs, we have observed that the app slows down remarkably due to R being an on-the-fly interpreted language. Moreover, functional graphs with > 250 MAGs (with individual colors) become less useful/interpretable, and we advise our users to rather employ parts of the ViMO code to their data locally to better optimize the parameters to fit the data. The code is freely available under GLP3 at https://github.com/magnusarntzen/ViMO.

### Alternative optimized workflow for metagenomics analysis in Galaxy

As metatranscriptomics and metaproteomics are mapped to, and thus depend on the quality of the metagenomic data, it is critical that this step is optimized using the best method available. The optimized MetaG workflow contains both a co-assembly (Fig. 4.4, 4.5), similar to the standard MetaG workflow above, but also with individual assemblies ran in parallel. For the individual assemblies, *trimmed* paired-end reads (Fig. 4.3) are *split* (Fig. 4.6) using the sample name as an element identifier into smaller collections per sample, containing the forward and reversed reads for each sample. Each sample is then *assembled* (Fig. 4.7) by MEGAHIT with k-mer sizes of 21, 29, 39, 59, 79, 99, 119, and 141, and the quality for assemblies are analyzed with *QUAST* in meta-mode (Fig. 4.9). The contigs are then *binned* (Fig. 4.8) by MaxBin2 (contig length ≥ 1000) and MAGs from each sample are *merged* (Fig. 4.10) together with the co-assembly into one collection with a sample identifier to trace the sample origin of the MAG in further downstream analysis. The merging of MAGs is followed by *dereplication* (algorithm ANImf, P_ani: 0.90, S_ani: 0.95) with dRep [32] (Fig. 4.11) for identification of groups of highly similar genomes and choosing the best representative genome within the genome sets. Completeness, contamination, and strain heterogeneity of each MAG is then reported by *CheckM* and read coverage by *CoverM* (Fig. 4.12). Further downstream analysis involves, as in MetaG, the prediction of nucleotide sequences and ORFs by FragGeneScan and functional annotation by InterProScan and dbCAN-HMMER. The predicted ORFs and nucleotide sequences are further used in the MetaP and MetaT workflow (Fig. 1).

Table 2 shows the contig counts and dataset statistics obtained by using both the standard and optimized MetaG workflows, on both the small bioreactor dataset used for developing these workflows, and for an in-house large complementary (Comp) dataset with 253 MAGs to stress-test the analysis pipelines.

**Table 2:** Contigs and dataset statistics for the two MetaG workflows

Dataset	Workflow	Contigs (MEGAHIT)	Unbinned contigs (%)	N50/L50	Longest contig
*Bioreactor*, Small dataset with 10 MAGs	MetaG	11,386	6	38,958/118	391,662
	Optimized MetaG		5		
	*Co-assembly*	11,296		28,943/145	351,556
	*Individual assemblies:*				
	*Sample-1*	4003		44,326/58	391,662
	*Sample-2*	12,098		27,635/128	391,715
*Comp*, Large dataset with 253 MAGs	MetaG	1,923,986	11	2309/93,659	797,197
	Optimized MetaG		20		
	*Co-assembly*	2,331,350		2474/10,2387	1,098,235
	*Individual assemblies:*				
	*Sample-1*	310,224		2109/14,283	625,541
	*Sample-2*	511,518		2530/24,745	715,289
	*Sample-3*	450,745		2083/20,003	872,994
	*Sample-4*	532,077		2820/21,306	862,734
	*Sample-5*	303,656		2548/13,484	497,688
	*Sample-6*	223,130		2523/9460	1,098,235

Contigs were analyzed with CoverM and metaQuast. For the optimized MetaG workflow, which includes both co- and single assemblies, the percentage of unbinned contigs is reported as the average number after dereplication. Both a small (bioreactor) and a large (in-house complementary; comp) dataset is included to stress-test the analysis pipelines

Contigs with similar tetranucleotide frequencies are binned to one MAG [56], and as is evident from Tables 2 and 3, the extra contigs provided by the individual assemblies in the optimized MetaG workflow, aids in the binning process and increases the number of high-quality MAGs compared to the bare use of co-assembly in the standard MetaG workflow.

**Table 3:** Quality of MAGs generated in the two workflows

MAG quality count	Bioreactor		Comp	
	MetaG	Optimized MetaG	MetaG	Optimized MetaG
Low^a^	6	0	172	42
Medium^b^	3	1	63	51
High^c^	1	6	18	50
Sum	10	7	253	143

The number of MAGs with low, medium, and high quality are counted for the standard and optimized MetaG workflow for both the Bioreactor and the Comp dataset

^a^< 50% completion, ≥ 10% contamination

^b^≥ 50% completion, < 10% contamination

^c^> 90% completion, < 5% contamination

The optimized MetaG workflow results in 10 MAGs from the co-assembly and 11 MAGs from the individual assemblies, from which 7 MAGs of almost exclusively high-quality are selected after the dereplication process (Table 4), whereas from the standard MetaG workflow, only one MAG is of high-quality.

**Table 4:** Taxonomy and quality values for MAGs generated with the two workflows

MetaG (Bin)	Taxonomy	Completeness	Contamination	Strain heterogeneity
Bin1	*Hungateiclostridium*	87.72	24.24	0.00
Bin2	*Coprothermobacter proteolyticus*	25.00	0.00	0.00
Bin3	*Coprothermobacter proteolyticus*	14.61	4.55	100.00
Bin4	*Coprothermobacter proteolyticus*	23.38	10.96	48.15
Bin5	*Acetomicrobium*	97.41	14.66	100.00
Bin6	*Coprothermobacter proteolyticus*	9.25	0.00	0.00
Bin7	Firmicutes	97.90	6.53	0.00
Bin8	*Methanothermobacter*	100	1.29	0.00
Bin9	Clostridia	98.08	8.44	0.00
Bin10	*Thermoclostridium stercorarium*	83.92	6.29	0.00
*Optimized MetaG*				
Opt-Bin1	*Hungateiclostridium thermocellum*	99.33	0.00	0.00
Opt-Bin2	*Coprothermobacter proteolyticus*	100.00	1.79	0.00
Opt-Bin3	*Acetomicrobium*	97.46	1.69	100.00
Opt-Bin4	*Tepidanaerobacter*	98.08	7.69	0.00
Opt-Bin5	Firmicutes	97.90	4.55	0.00
Opt-Bin6	*Methanothermobacter*	100.00	3.69	29.41
Opt-Bin7	*Thermoclostridium stercorarium*	98.60	4.06	0.00

Quality values were obtained by CheckM and taxonomic annotation by the program ‘CAT bins’. The data is from the Bioreactor dataset

**Fig. 4:**
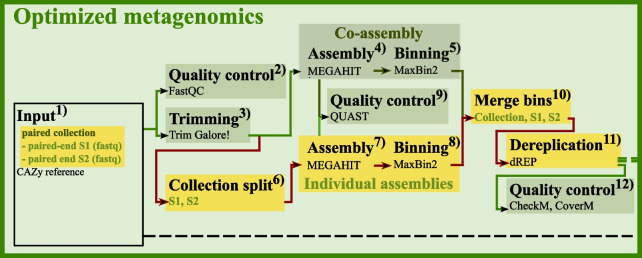
Optimized metagenomic workflow. We have created an optimized MetaG workflow to improve the quality of the MAGs. This is achieved by assembly and binning of the reads individually, in parallel to a co-assembly, and combined and dereplicated to exclude redundant MAGs before bin annotation, gene prediction and functional annotation. Two samples S1 and S2 are shown as an example. Differences to the original MetaG workflow are highlighted in yellow

Completeness and contamination of the MAGs are highly valuable metrics for the reliability of reconstructed metabolic pathways and annotated taxonomy [57]. In order to obtain at least “good-quality” MAGs (completeness > 70% and contamination < 10%) based on the standards by Bowers et al. [58], Galaxy currently contains three tools for this purpose: Binning_refiner [59], DAS Tool [60], and dRep. Binning_refiner searches for common contigs between each set of MAGs from different binning iterations creating the refined MAG, resulting in a non-redundant set of MAGs with decreased contamination and increased completeness [59]. Redundant MAGs lead to misinterpretations of the relative abundance and population dynamics throughout the different samples [61], a problem that is also addressed by DAS Tool and dRep. DAS Tool refines MAGs by evaluating the common contig set between MAGs, again obtained by different binning iterations, and the remaining potential MAGs are selected based on the F1-score followed by an iterative selection of high-scoring MAGs [60]. Another approach to extract only one high-quality representative of a replicate set of MAGs is dereplication by dRep using the MASH- and gANI algorithms to estimate distance and similarity between the MAGs and taking preset completion and contamination scores into account [32]. Dereplication results in a set of at least “good-quality” MAGs, which improves the downstream annotations and is therefore an important tool in our optimized MetaG workflow (Fig. 4).

## Concluding remarks

Herein we have presented the development of three integrated workflows for the analysis of meta-omics data, including a new tool for data visualization, ViMO. The workflows have been developed using a small dataset containing 10 MAGs, a subset of this is also provided as example input in the online version of ViMO. In addition, we have verified the workflows’ applicability to a larger dataset, as exemplified in Tables 2 and 3. Together, these Galaxy-based workflows and interactive visualizations allows scientists to explore and characterize microbiomes without prior knowledge in the use of compute clusters and scripting. Although nesting software in workflows promotes reproducible science, biological samples naturally vary in their complexity and heterogeneity, and may require different tool parameters. We therefore recommend that as our workflows are adapted by the wider community, each step in the workflows are adjusted and parameters optimized before analyzing new sample material. Our workflows may also be further extended with new capabilities from existing microbiome research tools [62] or as new tools are added to the Galaxy Platform in the future, such as for example FragPipe [53] for enhanced proteomics analysis, and Prodigal [63] for predicting genes in the MetaG workflow.

## Data Availability

The metagenomics, metatranscriptomics, and metaproteomics workflows developed herein are shared publicly within Galaxy; alternatively, they can be accessed via these links: https://usegalaxy.eu/u/mgnsrntzn/w/metagextended, https://usegalaxy.eu/u/mgnsrntzn/w/metap, https://usegalaxy.eu/u/mgnsrntzn/w/metat, ViMO is accessible online at https://magnusarntzen.shinyapps.io/VisualizerForMetaOmics/ and the source code is available at https://github.com/magnusarntzen/ViMO under the GPL-3 license. For the bioreactor data used in the development of Galaxy workflows and ViMO, metagenomics and metatranscriptomics sequencing reads are available in the sequence read archive under SRP134228, with specific numbers listed in Supplementary Table 6 in Kunath et al. [29]. The proteomics data for the same dataset is available in ProteomeXchange/PRIDE [64] with the data set identifier PXD016242.

## Abbreviations

MetaG: Metagenomics
MetaT: Metatranscriptomics
MetaP: Metaproteomics
MAG: Metagenome-assembled genomes
CAZymes: Carbohydrate-active enzymes
ORFs: Open reading frames
Mcf: Module completion fraction
